# A chromosome-level genome assembly of Korean mint (*Agastache rugosa*)

**DOI:** 10.1038/s41597-023-02714-x

**Published:** 2023-11-10

**Authors:** Hyun-Seung Park, Ick Hyun Jo, Sebastin Raveendar, Nam-Hoon Kim, Jinsu Gil, Donghwan Shim, Changsoo Kim, Ju-Kyung Yu, Yoon-Sup So, Jong-Wook Chung

**Affiliations:** 1 Department of Integrative Biological Sciences and Industry, Convergence Research Center for Natural Products, Sejong University, Seoul, 05006 Korea; 2 Department of Crop Science and Biotechnology, Dankook University, Cheonan, 31116 South Korea; 3 Department of Industrial Plant Science and Technology, Chungbuk National University, Cheongju, South Korea; 4 Phyzen Co.,Ltd, Seongnam, South Korea; 5 Department of Biological Sciences, Chungnam National University, Daejeon, South Korea; 6 Department of Crop Sciences, Chungnam National University, Daejeon, South Korea; 7 Department of Crop Science, Chungbuk National University, Cheongju, South Korea

**Keywords:** Plant sciences, Genome

## Abstract

*Agastache rugosa*, also known as Korean mint, is a perennial plant from the Lamiaceae family that is traditionally used for various ailments and contains antioxidant and antibacterial phenolic compounds. Molecular breeding of *A. rugosa* can enhance secondary metabolite production and improve agricultural traits, but progress in this field has been delayed due to the lack of chromosome-scale genome information. Herein, we constructed a chromosome-level reference genome using Nanopore sequencing and Hi-C technology, resulting in a final genome assembly with a scaffold N50 of 52.15 Mbp and a total size of 410.67 Mbp. Nine pseudochromosomes accounted for 89.1% of the predicted genome. The BUSCO analysis indicated a high level of completeness in the assembly. Repeat annotation revealed 561,061 repeat elements, accounting for 61.65% of the genome, with *Copia* and *Gypsy* long terminal repeats being the most abundant. A total of 26,430 protein-coding genes were predicted, with an average length of 1,184 bp. The availability of this chromosome-scale genome will advance our understanding of *A. rugosa*’s genetic makeup and its potential applications in various industries.

## Background & Summary

*Agastache rugosa*, a perennial plant belonging to the Lamiaceae family, is widely distributed in Korea, China, Taiwan, and Japan. In Korean traditional medicine, the aerial part of *A. rugosa*, known as “Gwakyang”, is prescribed for various ailments, such as miasma, cholera, anorexia, and vomiting^1^. *A. rugosa* produces phenolic compounds such as rosmarinic acid, which has antioxidant and antibacterial properties^2–5^. In addition to its uses in traditional herbal medicine, *A. rugosa* leaves are used as a spice or vegetable and its flowers as a tea ingredient^6^. Desta *et al*. assessed the antioxidant activity of various parts of *A. rugosa*—including the flowers, leaves, stems, and roots—and found that the leaves, flowers, and roots exhibited notably strong antioxidant properties^7^.

Previous research on *A. rugosa* has primarily concentrated on its secondary metabolites^3,4^, phenylpropanoid-biosynthetic genes^8–10^, and cell culture^11,12^. To date, there are no whole genome sequences available for *A. rugosa*, and only transcriptome data have been published^13^. An integrated analysis of its metabolites and genome will provide insight into chemotype breeding of *A. rugosa* and improve its economic value in the market.

In this study, we assembled the chromosome-level genome of *A. rugosa* using Nanopore sequencing and Hi-C technology. The final genome assembly had a scaffold N50 of 52.15 Mbp, totaling 410.67 Mbp. With integration of Hi-C data, nine pseudochromosomes were generated, accounting for 89.1% of the entire predicted genome. The first chromosome-scale genome of *A. rugosa* provides a foundational genetic resource for breeding programs targeting enhanced production of secondary metabolites like rosmarinic acid and essential oils. This genome assembly bolsters the efficiency of genotyping methods such as GBS, facilitating more precise QTL analysis or GWAS, which are crucial for optimizing agricultural traits.

## Methods

### Sampling and sequencing

A breeding line, AG34, of *A. rugosa*, sourced from a specific population in the field, was chosen for reference genome sequencing and assembly. This line was derived from original natural accessions obtained from the Chungbuk National University (Korea). Young leaf samples were collected once during the vegetative stage after being grown in a greenhouse for three months. Leaf tissue samples were stored at −80 °C and used for DNA extraction, whole genome sequencing, and Hi-C library construction. DNA was extracted using the Biomedic Plant gDNA extraction kit (#BM20211222A, Korea) following the manufacturer’s instructions.

An Oxford Nanopore Technology (ONT) sequencing library was constructed using the ONT genomic ligation sequencing kit SQK-LSK110 (ONT, UK). ONT sequencing was performed using the flow cell vR9.4 (FLO-MIN106) and GridION platform operated with MinKNOW Core 4.4.3 following the manufacturer’s instructions. We obtained 55.9 Gb of raw genomic data. Guppy v5.0.17, embedded in MinKNOW^14^, was used to convert raw ONT sequencing data (FAST5 files) to FASTQ format using the default parameters of the high-accuracy method. All ONT sequencing procedures were conducted by Phyzen Co. (www.phyzen.com, Korea). Paired-end (PE) Illumina sequencing was also conducted with the NovaSeq6000 platform after constructing a standard Illumina paired-end library. We obtained 115.5 Gb of raw data from Illumina sequencing.

Total RNA was extracted from leaf tissue of the same material used for the genome sequencing of *A. rugosa*, and the transcriptome was sequenced on the Illumina NovaSeq6000 platform by Macrogen Co. (www.macrogen.com, Korea). The RNA reads were used for gene annotation.

### Sequence trimming and genome size estimation

**Fig. 1:**
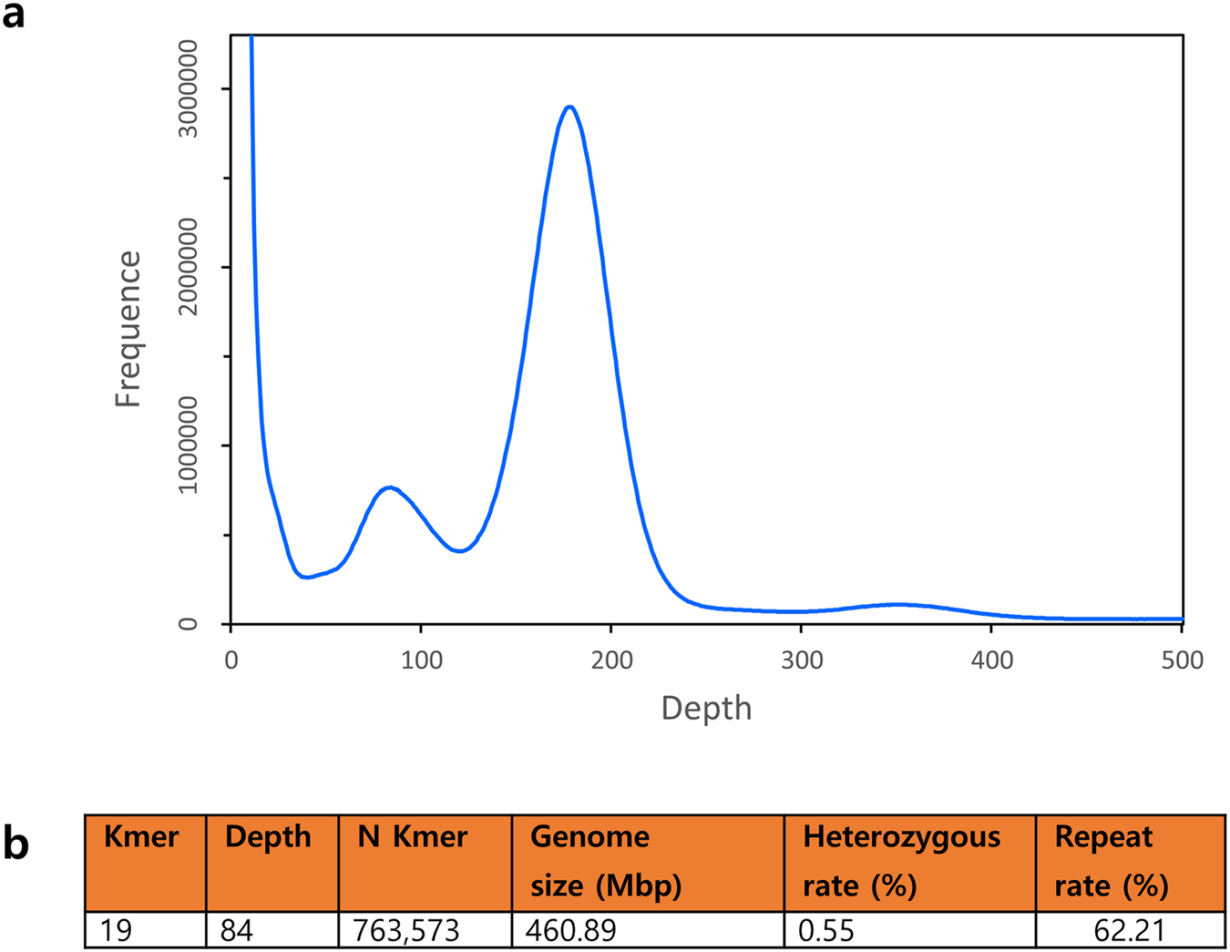
The result of K-mer analysis. (**a**) 19-mer frequency distribution in *A. rugosa* genome. The X-axis is the k-mer depth, and Y-axis represents the frequency of the k-mer for a given coverage. (**b**) Statistics of K-mer analysis.

ONT data were trimmed using Porechop (v.0.2.3, https://github.com/rrwick/Porechop) with default parameters to remove adaptors and chimeric sequences. Raw Illumina sequencing data were trimmed using fastp (v.0.21.0, https://github.com/OpenGene/fastp) with default parameters. The amount of trimmed Illumina PE sequencing data was 97 Gb, which was used for further genome size estimation based on k-mer analysis. An optimal k-mer value of 19 was calculated by Jellyfish (v2.0)^15^, and the genome size was estimated using GenomeScope (v2.0)^16^. The estimated genome size of *A. rugosa* based on k-mer analysis was 460.89 Mbp, which is slightly smaller than the 520 Mb previously reported using flow cytometry^17^. The heterozygous rate was 0.55%, and the repeat rate was 62.21% (Fig. 1).

### Contig assembly

The first round of *de novo* assembly was performed using NextDenovo assembler (v.2.3.1, https://github.com/Nextomics/NextDenovo) with default parameters, employing only preprocessed 55,923,595,489 bp of ONT data(~121X of estimated genome size, 460Mbp). Assembled contigs were then polished using NextPolish (v1.3.1, https://github.com/Nextomics/NextPolish) with trimmed Illumina PE sequencing data. Haplotigs were removed using Purge Haplotigs^18^ with default parameters. The assembly statistics improved, with fewer contigs and increased minimum, average contig lengths, and N90 (see Table S1). Finally, a draft genome assembly was generated with 221 contigs totaling 410.65 Mbp, with a contig N50 of 3.85 Mbp (Table 1).

**Table 1:** Assembly statistics of *A. rugosa*.

***De novo*** **assembly**	
Total contigs number	221
Total size of assembled contigs (bp)	410,656,262
Minimum length of contig (bp)	48,164
Maximum length of contig (bp)	12,657,832
Average length of contigs (bp)	1,858,173
Contig N50 (bp)	3,851,190
Contig N90 (bp)	885,118
GC contents (%)	36.51
**Final statistics of Hi-C scaffolding**	
The number of scaffolds (pseudomolecule)	9
Unscaffolded contigs	21
Total length	410,677,362
Total length of scaffolds anchored to chromosomes	405,296,100
Total length of unscaffolded contig	5,381,262
Maximum length of unscaffolded contigs	697,320
Minimum length of scaffold	70,820
Maximum length of scaffold	73,606,202
Scaffold N50	52,151,255
Scaffold N90	32,072,577

### Chromosome-level genome assembly using Hi-C data

**Fig. 2:**
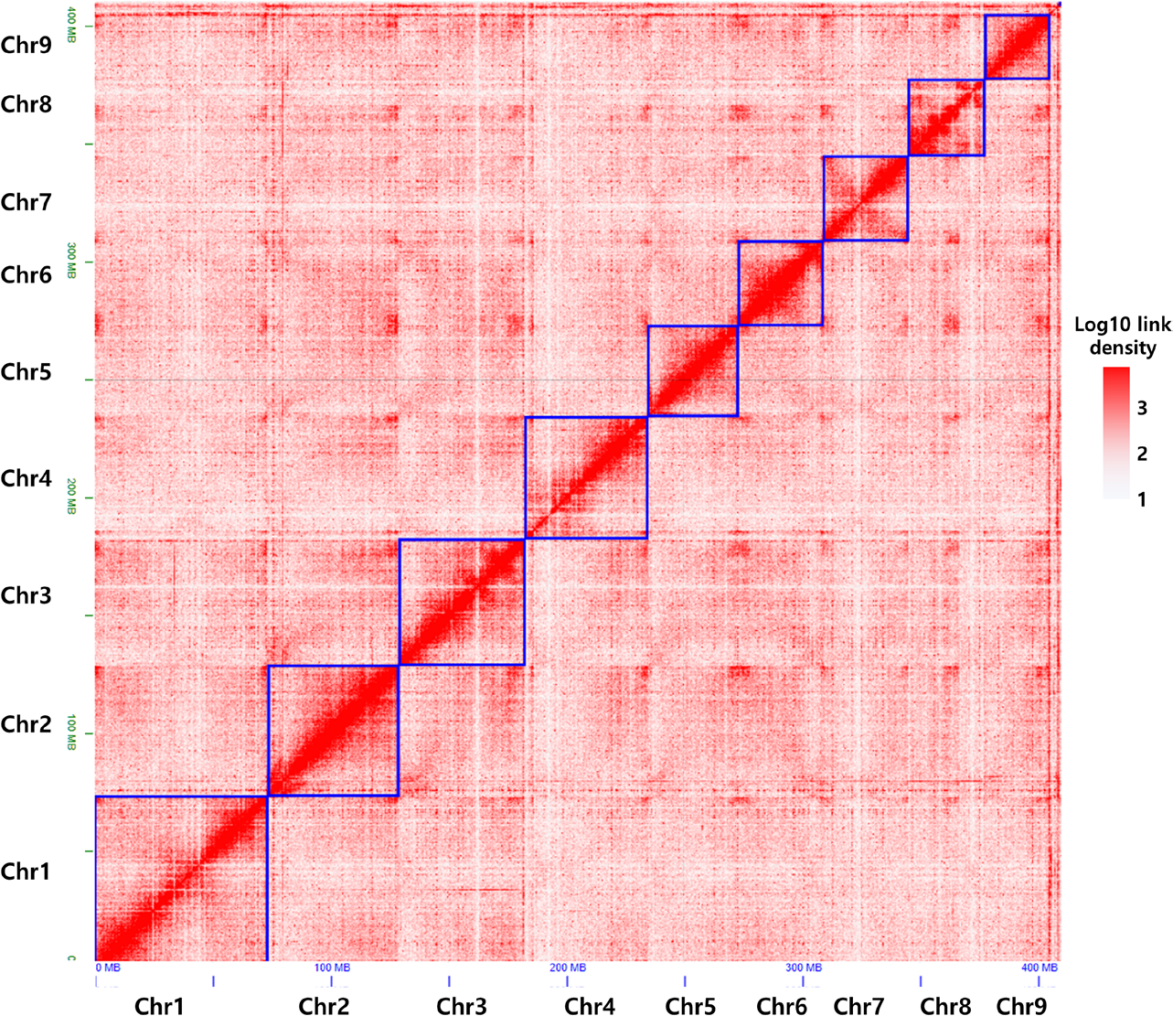
Hi-C contact map the chromosome-level assembly of *A. rugosa*. The intensity of interactions was calculated using a bin size of 140 K.

**Fig. 3:**
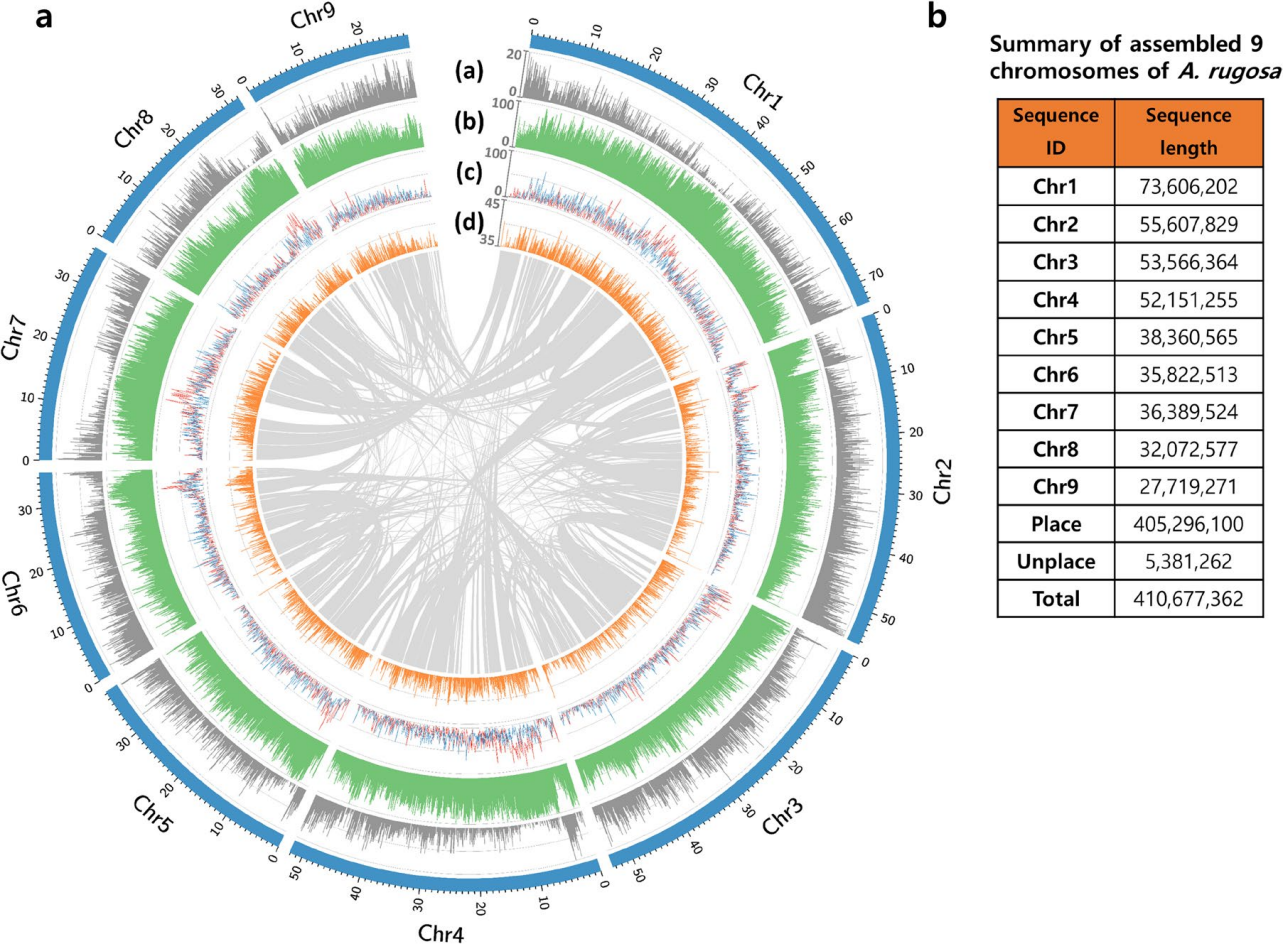
Overview of genome features of the *A. rugosa*. Syntenic block among inter-chromosome were analyzed with MCScanX. (**a**) Gene distribution, (**b**) Repeat percentage(%), (**c**) Gypsy (red line) and Copia (blue line) LTR distribution (%), (**d**) GC content(%).

A Hi-C library of *A. rugosa* was constructed for chromosome assembly using the Proximo^TM^ Hi-C Plant Kit (Phase Genomics, United States) following the manufacturer’s instructions. A total of 30.77 Gbp of clean Hi-C data were generated and aligned to the assembled contigs using BWA-MEM (v0.7.17)^19^ with -5SP and -t 8 options specified. Chromosome-level scaffolding was performed with the Phase Genomics Proximo Hi-C genome scaffolding platform based on the LACHESIS method^20^, and sequences were anchored to nine pseudochromosomes with chromosome lengths ranging from 27.7 Mb to 73.6 Mb. Our chromosome-scale assembly coincides with that from a previous karyotype analysis, as the base chromosome number of *Agastache* species is reported to be nine, and *A. rugosa* is a diploid species^21,22^. Additional manual correction of the chromatin contact matrix was performed using Juicebox (https://github.com/aidenlab/Juicebox). The nine pseudochromosomes were clearly identified by distinct interaction signals in the Hi-C interaction heatmap (Fig. 2), and the final assembled genome was 410.68 Mbp, with a scaffold N50 of 52.15 Mb, accounting for 89.1% of the predicted genome size based on the k-mer analysis (Table 1 and Fig. 3). The assembled genome sizes of Lamiaceae species show a wide range of variation: *A. rugosa* in this study (410.68 Mbp), *Perilla frutescens* var. *hirtella* (676.94 Mbp)^23^, *P. frutescens* var. *frutescens* (1.2 Gbp)^23^, *Salvia hispanica* (321.47 Mbp)^24^, and *Salvia splendens* (805.9 Mbp)^25^.

### Assessment of the genome assemblies

The completeness of the assembled genome was evaluated using BWA-MEM (v0.7.17)^19^ and Benchmarking Universal Single-Copy Orthologs (BUSCO, v5.2.1)^26^ with the embryophyta_odb10 lineage dataset. Approximately, 98.04% of the Illumina short read were aligned to genome, of which 89.6% of reads were properly mapped. The BUSCO analysis showed that the assembled draft genome sequence contained 1,596 (98.9%) complete BUSCOs, including 1,533 (95.0%) single-copy BUSCOs, 63 (3.9%) duplicated BUSCOs, and 7 (0.4%) fragmented BUSCOs (Table 2).

**Table 2:** Result of the BUSCO assessment of *A. rugosa*.

Type	Genome	
	Count	Ratio (%)
Complete BUSCOs (C)	1,596	98.9
Complete and single-copy BUSCOs (S)	1,533	95.0
Complete and duplicated BUSCOs (D)	63	3.9
Fragmented BUSCOs (F)	7	0.4
Missing BUSCOs (M)	11	0.7
Total BUSCO groups searched	1,614	100.0

### Repeat annotation

The *de novo* repeat families were identified with RepeatModeler^27^, and by LTR_retriever^28^, then repetitive sequences were masked using RepeatMasker 4.0.9 (http://www.repeatmasker.org). A total of 561,061 repeat elements were identified, accounting for 61.65% of the *A. rugosa* genome. Among the various repeat elements, *Copia* and *Gypsy*, which are long terminal repeats (LTRs), were dominant in the genome, accounting for 14.98% and 13.91%, respectively (Table 3).

**Table 3:** Repetitive elements annotation in *A. rugosa*.

Class	Number of elements	Sequence length (bp)	Percentage of genome (%)
**DNA**	37,867	10,748,442	2.62%
CMC-EnSpm	3,533	2,472,316	0.68%
MULE-MuDR	10,783	9,767,543	2.38%
PIF-Harbinger	5,955	2,660,594	0.65%
TcMar-Pogo	646	104,439	0.03%
TcMar-Stowaway	578	510,816	0.12%
hAT-Ac	3,793	2,366,869	0.58%
hAT-Tag1	477	202,820	0.05%
hAT-Tip100	776	289,420	0.07%
**LINE**	1,973	252,866	0.06%
L1	3,602	1,630,673	0.40%
**LTR**	48,566	12,283,572	2.99%
Caulimovirus	5,413	10,430,421	2.54%
Copia	34,703	61,518,387	14.98%
Gypsy	41,357	57,125,275	13.91%
unkown	27,449	15,268,074	3.72%
**RC**	—	—	—
Helitron	5,338	2,634,800	0.64%
**SINE**	5,191	1,120,846	0.27%
**tRNA**	157	42,282	0.01%
**Unknown**	229,870	57,817,026	14.08%
**total interspersed**	468,027	249,247,481	60.69%
**Low_complexity**	16,665	793,497	0.19%
**Simple_repeat**	76,369	3,132,257	0.76%
**Total**	561,061	253,173,235	61.65%

### Gene prediction and annotation

Gene prediction involved a combination of evidence-based annotation methods and *ab initio* prediction using repeat-masked assembly sequences. RNA-Seq data were assembled by Trinity and used for the transcript set. Additionally, protein data from four related Lamiaceae species were obtained from the NCBI. The first round of gene prediction was performed using MAKER (v3.01.03)^29^ with evidence data, the transcript set and the protein data from the four related species. The *ab initio* gene predictions were conducted on only the first gene models with sufficient evidence (AED of 0.25 or less) using GeneMark-ES (v4.38)^30^, SNAP (v2006-07-28)^31^, and Augustus (v3.3.2)^32^. Final gene predictions were confirmed again based on the first gene model and *ab initio* gene model using MAKER3 (v3.01.03)^29^ and EvidenceModeler (v1.1.1)^33^. In total, 26,430 protein-coding genes were predicted and annotated, with an average gene length of 1,184 bp (Table 4). The complete BUSCOs of predicted gene set were calculated as 98.9%.

**Table 4:** Summary of gene annotation.

Type		Number	Percent
**BLASTP (DIAMOND)**	NCBI nr	24,583	93.01
	Araport11	21,770	82.37
**Protein domains (InterProScan)**		20,523	77.65
**Gene Ontology (BLAST2GO)**		14,946	56.55
**KEGG pathway (KAAS webtools)**		10,047	38.01
**Annotated genes**		24,624	93.17
Total length of genes (bp)		31,296,426	
Smallest gene length (bp)		102	
Largest gene length (bp)		15,765	
Average gene length (bp)		1,184	
GC content (%)		46.61	
**Unannotated**		1,847	6.99
**Total number of genes**		26,430	

**Fig. 4:**
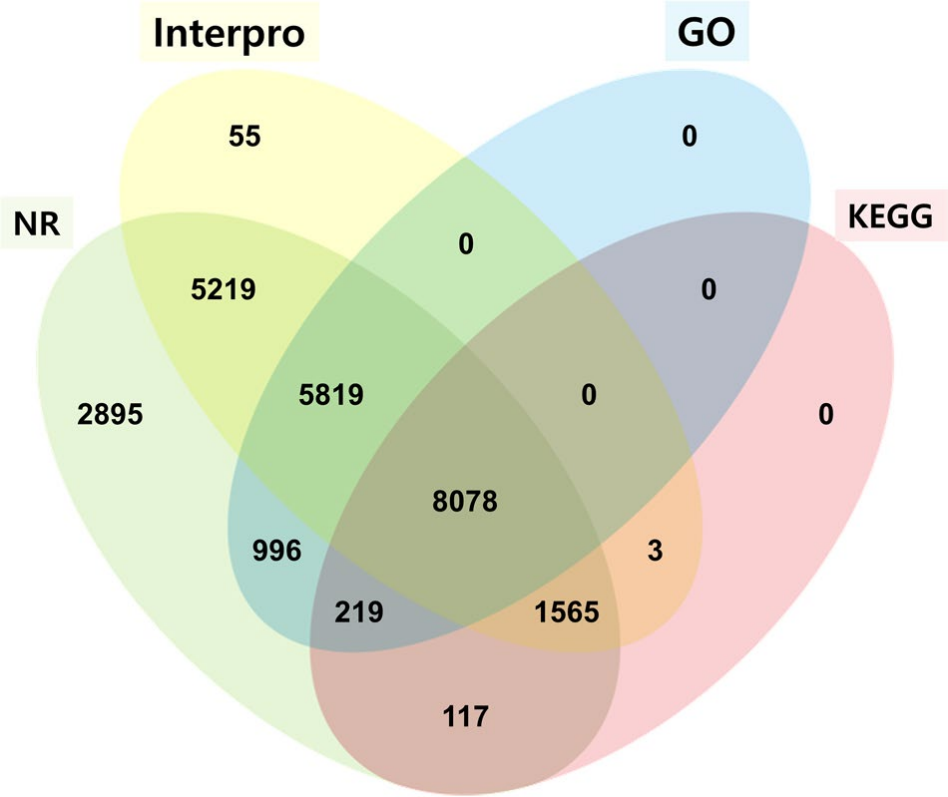
Venn diagram of the number of genes from *A. rugosa* with homology or functional classification using multiple public databases.

The predicted genes of *A. rugosa* were functionally annotated by comparing their similarities against those in the NCBI nonredundant (nr) protein database and the reference genome Araport11 of *Arabidopsis thaliana* using DIAMOND (v0.9.30.131)^34^ with an E-value cutoff of 1E-5. Conserved protein domains were predicted by InterProScan (v5.34-73.0)^35^. Gene Ontology analysis was conducted using the Blast2GO command line (v.1.4.4), and genes were assigned to metabolic pathways by comparing them to those in the Kyoto Encyclopedia of Genes and Genomes (KEGG) pathway database^36^ using the KEGG Automatic Annotation Server (KAAS) webtools (v2.1)^37^. A total of 24,624 genes were successfully annotated for *A. rugosa*, accounting for 93.2% of all predicted genes (Table 4 and Fig. 4). Predicted gene models were comparable to four other Lamiaceae species in aspects such as gene count, average CDS length, average exons per gene, and average exon and intron length (Table 5).

**Table 5:** The comparison of the gene models annotated from *A. rugosa* genome and other Lamiaceae.

Species (Accession number in GenBank)	Gene Number	Average CDS length	Average exons per gene	Average exon length	Average intron length
*Agastache rugosa* (GCA_031470985.1)	26,867	1,177	5.21	226.01	405.90
*Perilla frutescens* var. *frutescens* (GCA_019511825.2)	38,941	1,259	5.19	242.42	395.75
*Perilla frutescens* var. *hirtella* (GCA_019512045.2)	23,675	1,252	5.08	246.41	398.23
*Salvia hispanica* (GCF_023119035.1)	36,995	1,379	9.20	277.52	42.18
*Salvia splendens* (GCF_004379255.1)	64,211	1,391	10.54	276.74	27.35

### Ortholog and phylogenetic analysis

**Fig. 5:**
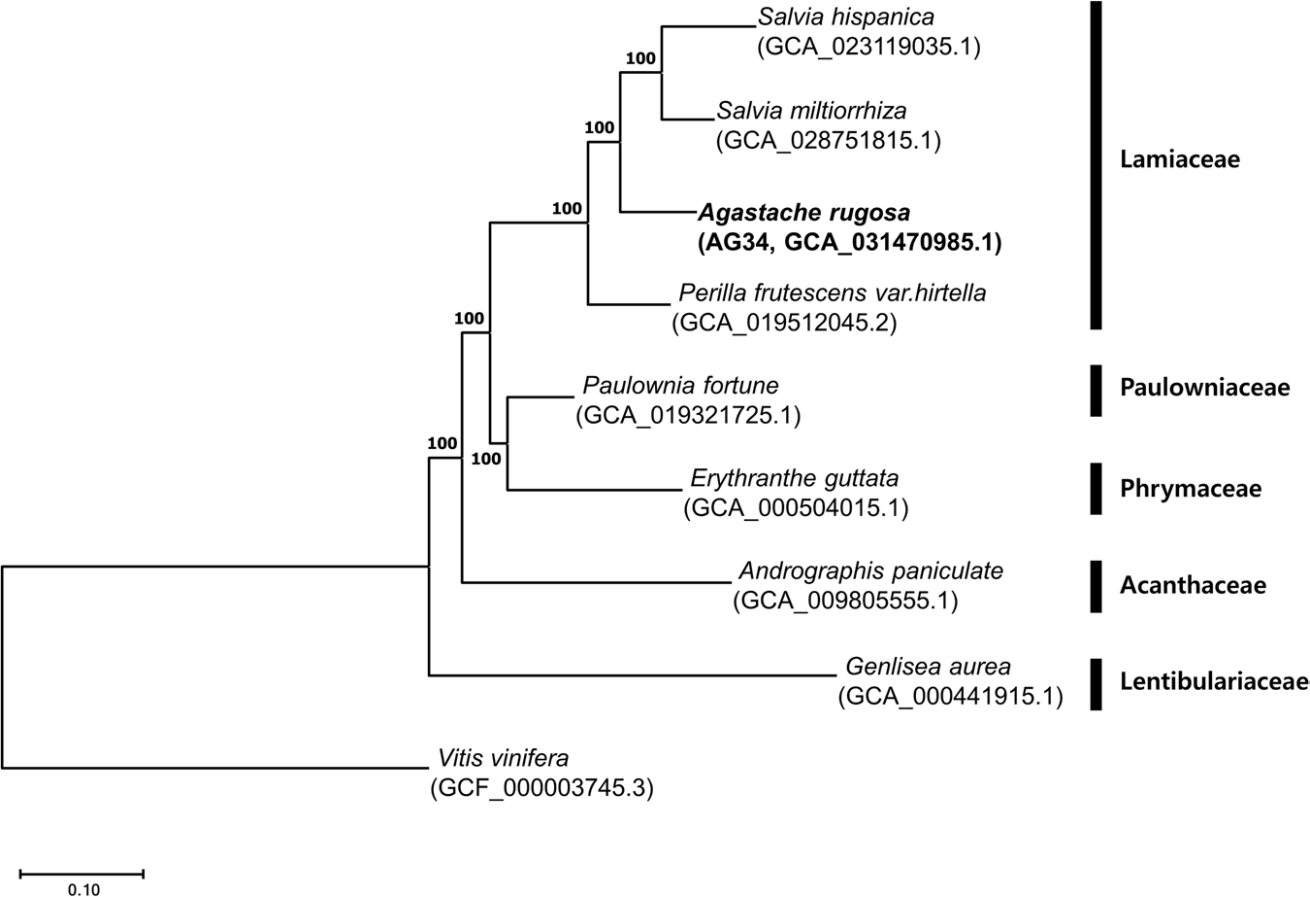
Phylogenetic relationship of Lamiales species.

Orthologs between *A. rugosa* and eight other plants (seven from the order Lamiales: *S. hispanica*^24^*, Salvia miltiorrhiza*^38^*, P. frutescens* var. *hirtella*^23^, *Paulownia fortune*^39^*, Erythranthe guttata*^40^*, Andrographis paniculata*^41^, and *Genlisea aurea*^42^, along with one outgroup, *Vitis vinifera*^43^) were identified using OrthoFinder (v2.5.4)^44^. The sequences for these plants were sourced from the NCBI database (http://www.ncbi.nlm.nih.gov/). From these, 371 single-copy orthologous genes were extracted, concatenated, and aligned using the Multiple Alignment program for amino acid or nucleotide sequences (MAFFT)^45^. We then constructed a maximum likelihood phylogenetic tree of these orthologous genes using RAxML (v8.2.12)^46^ under the JTT model, Gamma Distributed With Invariant Sites (G + I), with a bootstrap value of 1000. Four species, namely *A. rugosa, S. hispanica, S. miltiorrhiza*, and *P. frutescens* var. *hirtella*, all of which belong to the Lamiaceae family, clustered in the same clade. Notably, *A. rugosa* exhibited a closer relation to the two *Salvia* species (Fig. 5). These findings are consistent with previous phylogenetic studies based on the chloroplast genome^47^.

## Data Records

The genomic Illumina sequencing data were deposited in the Sequence Read Archive at the NCBI (SRR24282004)^48^.

The genomic Nanopore sequencing data were deposited in the Sequence Read Archive at the NCBI (SRR24282001)^49^.

The transcriptome Illumina sequencing data were deposited in the Sequence Read Archive at the NCBI (SRR24282003)^50^.

The Hi-C sequencing data were deposited in the Sequence Read Archive at the NCBI (SRR24282002)^51^.

The final chromosome assembly was deposited in GenBank at the NCBI (GCA_031470985.1)^52^.

The annotation result of gene structure, functional prediction, and final chromosome assembly were deposited in the Figshare database (10.6084/m9.figshare.22730084)^53^.

## Technical Validation

The integrity and concentration of the extracted DNA and RNA were assessed with a TapeStation 2200 and an Agilent 2100 Bioanalyzer (Agilent Technologies, CA, USA), respectively. In a comparative context, the complete BUSCO value for *A. rugosa* (98.9%) exceeds those of *P. frutescens* var. *frutescens* (92.7%)^23^, *P. frutescens* var. *hirtella* (92.5%)^23^, *S. splendens* (92.0%)^25^, and *S. hispanica* (97.8%)^24^, underscoring its relative completeness and quality within the Lamiaceae family.

### Supplementary information

Supplementary

## Data Availability

No in-house code or scripts were used in this study. Commands and pipelines used for data processing were executed using their corresponding default parameters.
