# Supplementary material for: A chromosome-level genome assembly of Korean mint (Agastache rugosa)
Source: Sci Data. 2023 Nov 10;10:792. doi: 10.1038/s41597-023-02714-x (PMC10638305; doi:10.1038/s41597-023-02714-x)
Supplement: Supplementary file 1 — Supplementary [file 41597_2023_2714_MOESM1_ESM.pdf]

Supplementary information

Type	Value
Contigs number	376
Contigs length (bp)	436,874,142
Min length (bp)	15,110
Max length (bp)	12,657,832
Average length (bp)	1,161,899
N50 (bp)	3,656,981
N90 (bp)	541,589
GC Ratio (%)	36.54

Table S1. Assembly statistics before Purge Haplotigs.
